# Accelerometer Cut-Points for Physical Activity Assessment in Adults with Mild to Moderate Huntington’s Disease: A Cross-Sectional Multicentre Study

**DOI:** 10.3390/ijerph192214834

**Published:** 2022-11-11

**Authors:** Lucía Simón-Vicente, Jéssica Rivadeneyra-Posadas, María Soto-Célix, Javier Raya-González, Daniel Castillo, Sara Calvo, Carla Collazo, Alejandro Rodríguez-Fernández, Vitoria S. Fahed, Natividad Mariscal, Álvaro García-Bustillo, Laura Aguado, Esther Cubo

**Affiliations:** 1 Burgos University Hospital, 09006 Burgos, Spain; 2 Faculty of Health Sciences, University of Burgos, 09001 Burgos, Spain; 3 Faculty of Health Sciences, University Isabel I, 09003 Burgos, Spain; 4 Valoración del Rendimiento Deportivo, Actividad Física y Salud y Lesiones Deportivas (REDAFLED), University of Valladolid, 42004 Soria, Spain; 5 VALFIS Research Group, Institute of Biomedicine (IBIOMED), Faculty of Sciences of Physical Activity and Sports, Universidad de Leon, 24004 León, Spain; 6 Insight Centre for Data Analytics, University College Dublin, D04 V1W8 Dublin, Ireland

**Keywords:** accelerometry, energy metabolism, rehabilitation, actigraphy, calorimetry, indirect

## Abstract

Accelerometers can estimate the intensity, frequency, and duration of physical activity in healthy adults. Although thresholds to distinguish varying levels of activity intensity using the Actigraph wGT3X-B have been established for the general population, their accuracy for Huntington’s disease (HD) is unknown. We aimed to define and cross-validate accelerometer cut-points for different walking speeds in adults with mild to moderate HD. A cross-sectional, multicentre, case-control, observational study was conducted with a convenience sample of 13 symptomatic ambulatory HD participants. The accelerometer was placed around the right hip, and a heart monitor was fitted around the chest to monitor heart rate variability. Participants walked on a treadmill at three speeds with light, moderate and vigorous intensities. Correlation and receiver operation curve analyses were performed between the accelerometer magnitude vector with relative oxygen and heart rate. Optimal cut-points for walking speeds of 3.2 km/h were ≤2852; 5.2 km/h: >2852 to ≤4117, and in increments until their maximum velocity: >4117. Our results support the application of the disease-specific cut-points for quantifying physical activity in patients with mild to moderate HD and promoting healthy lifestyle interventions.

## 1. Introduction

According to the World Health Organization (WHO) [1], physical inactivity is the fourth leading risk factor for mortality, accelerating decreased mobility and functional skills [2]. Scientific evidence shows that physical activity reduces the risk of coronary heart disease, stroke, type II diabetes mellitus, arterial hypertension, and osteoporosis [2] and might be beneficial in preventing and treating several neurodegenerative disorders such as Parkinson’s disease (PD) and Alzheimer’s disease [3,4]. In this regard, previous studies reported that in Huntington’s disease (HD), an autosomal dominant hereditary neurodegenerative disorder characterized by chorea and bradykinesia [5], cognitive impairment [6], and psychiatric disturbances [7], a sedentary lifestyle may lead to earlier disease onset and decreased quality of life [8].

Exercise and physical activity levels can be measured by specific methods that provide reliable and objective information. Accelerometers are devices capable of collecting data on the intensity, frequency, and duration of physical activity [9]. It is possible to derive physical activity information from accelerometers by observing the changes of acceleration in the body, which are collected over a period of time named epoch. This information reflects intensity, but to provide prognostic measures, it is necessary to translate it into energy expenditure calibrated against standardized activities [9]. These activity monitors have also been validated by indirect calorimetry and calibrated in metabolic activity equivalents (METs). Several investigations have been conducted with different populations to analyze the relationship between counts per minute and energy expenditure [10,11,12,13,14]. These studies aimed to estimate energy expenditure for physical activity and time spent at various absolute intensities of physical activity (light, moderate and vigorous) to establish specific cut-points. Although cut-points for determining the level of physical activity have been determined for other neurodegenerative diseases [10], translating these cut-points to HD may lead to misleading, inaccurate conclusions. In HD, the influence of difficulties on gait and walking performance due to hyperkinetic and hypokinetic movements might change the slope and/or strength of the association between activity counts and energy expenditure. Therefore, we aimed to define accelerometer cut-points for different walking speeds in adults with mild to moderate HD.

## 2. Material and Methods

### 2.1. Design

A cross-sectional, observational study was conducted at the Exercise Physiology, Health, and Quality of Life laboratory of the University Isabel I (Burgos, Spain). The research team was composed of Ph.D. Sports Scientists, Occupational Therapists, Movement Disorder Neurologist, and Ph.D. Nutrition Scientists. This study is registered on clinicalTrials.gov Identifier, with the registration number NCT05250323.

### 2.2. Participants

A convenience sample of symptomatic ambulatory HD patients was recruited from the Neurology Department of the Burgos University Hospital, a partner site for the European Enroll study (Enroll-HD), an international platform with annual clinical follow-up of HD patients. The inclusion criteria consisted of a confirmed genetic diagnostic of HD (≥36 CAG repeats in the HTT gene), motor symptoms manifestations with a score greater than 4 on the motor subscale of the Unified Huntington Disease Rating Scale (UHDRS) [15], ability to walk with minimal support and no sensory deficits or other systemic diseases in the investigator’s judgment that could interfere with the execution of the study. The exclusion criteria consisted of being under pharmacological treatment for diabetes mellitus, thyroid, suffering from other neurodegenerative, cardiac, pulmonary, or skeletal-muscular diseases, being pregnant or breastfeeding, having active cancer, or taking medication that could affect metabolism/endocrine function.

### 2.3. Ethics

The Research Ethics Committee approved this study of the University Hospital of Burgos and Soria. All the procedures were conducted in accordance with Good Clinical Practice standards and following the ethical principles established in the Declaration of Helsinki. Participants gave their written consent and were assigned a study code to anonymize their data. Information and data were stored in a secure folder in a secure location dedicated to this study. 

### 2.4. Procedure

#### 2.4.1. Test Protocol

The study was carried out in the Exercise Physiology, Health, and Quality of Life laboratory of the University Isabel I (Burgos, Spain). Prior to the visit, participants were instructed to attend the visit at least 3–5 h after breakfast, refrain from drinking alcohol or using nicotine 2 h before the visit and refrain from moderate or vigorous physical exercise for 24 h before the assessment. Participants signed the informed consent form and collected nutritional information, such as anthropometry, skinfolds, bioimpedance, and dynamometry.

Participants started the test in the supine position on a stretcher for 10 min. For the next part, participants climbed onto a treadmill and, after being fitted with a harness to avoid the risk of injury in the event of a fall, began the treadmill familiarization period. They walked at a constant intensity of 3.2 km/h with a constant gradient of 1% and 3% for three minutes. After a one-minute rest, they walked at a constant intensity of 5.2 km/h with a constant gradient of 1% and 3%. After three minutes of rest, the incremental test began. The participant started walking at 1.5 km/h with 0.5 km/h increments every minute until they reached their maximum natural velocity. An accelerometer was placed around the right hip using an elastic strap, and heart rate (HR) variability was determined using a specialized HR monitor fitted around the chest. Throughout the procedure, energy expenditure was measured by indirect calorimetry.

#### 2.4.2. Assessments

Caloric intake was then determined by recording dietary habits using the SUN Food Frequency Questionnaire and a three-day food diary [16]. All HD participants were evaluated at baseline by a certified movement disorder neurologist using a standardized HD assessment tool, the UHDRS, including the motor subscale (UHDRS-TMS), with high scores denoting greater impairment [15]. Disease severity was assessed using the Total Functional Capacity (TFC) [15], with higher scores indicating more intact functioning. The severity of psychiatric symptoms was assessed using the Problem Behaviours Assessment (PBA), with higher scores indicating greater severity [17]. Cognition was screened using the Mini-Mental State Examination (MMSE) with a cut-off of 24, with lower scores indicating cognitive impairment [18].

#### 2.4.3. Accelerometers

The accelerometer used was the ActiGraph wGT3X-B model. It is a lightweight (19 g) and small (4.6 × 3.3 × 1.5 cm) device commonly used for objectively measuring physical activity. The accelerometer data consists of acceleration in three axes: vertical up and down (y), horizontal left and right (x), and horizontal forward and backward (z). We extracted the vector magnitude (VM), calculated as the square root of the sum of the squared points from each axis [13], and analyzed the accelerometer data as counts per minute. These data were collected in 60 s epochs through the Actilife software (v.6.13.49) and were later converted and exported as a spreadsheet for processing. To measure energy expenditure, we recorded the respiratory exchange using indirect calorimetry. The device consisted of an adaptive mask that was connected to a pneumotachograph (VO_2_) and gas analyzer (Medisoft Ergocard, Medisoft Group, Sorinnes, Belgium) on a breath-by-breath basis that determined oxygen consumption (VO_2_) and carbon dioxide production (CO_2_), giving respiratory exchange values. Participants breathed through the mask equipped with inspiratory valves that transmitted the O_2_ data to a computer for analysis [19]. This device was calibrated prior to testing according to the manufacturer’s instructions. We used a specialized device to monitor heart rate during the treadmill test (Polar Electro V800, Kempele, Finland, 76 g, 5.6 × 3.7 × 12.7 cm). In relation to the anthropometric measurements, we calculated the body mass index (BMI) following the formula weight (kg)/ height^2^ (m^2^). We classified the body mass index according to the International WHO standards considering BMI ≥ 18.5 < 25.0 kg/m^2^ normal, BMI < 18.5 kg/m^2^ underweight, BMI ≥ 25.0 < 30.0 kg/m^2^ overweight, and BMI ≥ 30.0 obesity [20]. 

### 2.5. Data Analysis

Descriptive statistics for participants and main outcomes are presented as the mean and standard deviation (SD) for continuous variables, the median, and the 25th–75th percentiles for non-normally distributed or ordinal data. The normality of the variables was evaluated using the Shapiro–Wilk test. We calculated the frequency distribution and percentages to describe categorical variables. Data were analyzed using SPSS version 25 for Windows (SPSS Inc., Chicago, IL, USA) and Microsoft Excel. The level of significance was set at *p* < 0.05.

For further analysis, physical activity was stratified into three categories: light, moderate, and vigorous. Light activity data consisted of the 3.2 km/h walking speed, the moderate activity of the 5.2 km/h walking speed, and the vigorous activity was considered the final trial, in which participants walked in increments until their maximum velocity. 

Accelerometer cut-points were identified with receiver-operating- characteristic (ROC)- curve analysis, where an area under the curve (AUC) of 0.5 indicates that the test is no better than chance, 0.6–0.7 poor, 0.7–0.8 fair, 0.8–0.9 good, 0.9–1 excellent while 1.0 indicates a perfect test. The optimal cut-point is the closest to the upper left corner of the ROC-curve figure, representing 100% sensitivity and 100% specificity. To identify the most appropriate sensitivity and specificity value as the cut-points, we used the distance to the upper left corner, Youden’s index [21]. Indices computed from these graphs provide an empirical basis for determining the most appropriate cut-point to minimize misclassification [22]. We used linear regression analysis to establish the relationship between the VM with VO_2_ and the VM with HR.

## 3. Results 

A total of 14 individuals with HD (50% females) were included in the study. One woman was excluded because she could not perform the protocol due to severe chorea. The mean age ± SD of the participants was 57.23 ± 9.98 years, weight 65.36 ± 10.78 kg, and mean height 161.07 ± 5.99 cm. The mean Body Mass Index was 25.29 ± 4.76 kg/m^2^, and the mean MMSE was 27.6 ± 2.1.

The median score of the UHDRS (TMS) analysis for the participants was 32.5 (interquartile range [IQR] 23.75–40) points, with a median TFC score of 9.5 (7.75–12.25). The HR measurements obtained for the different walking speeds were 107.31 ± 14.61 beats per minute (bpm) for a walking speed of 3.2 km/h, 116.47 ± 14.48 bpm for a walking speed of 5.2 km/h, and 124.82 ± 20.39 bpm for incremental walking speed. 

Table 1 shows generated cut-points for the lowest and highest walking speeds based on the ROC analysis and their respective AUC values. Consequently, for the walking speed of 3.2 km/h, estimated counts were ≤2852, for the 5.2 km/h between 2852–4117, and the incremental test > 4117.

**Figure 1:**
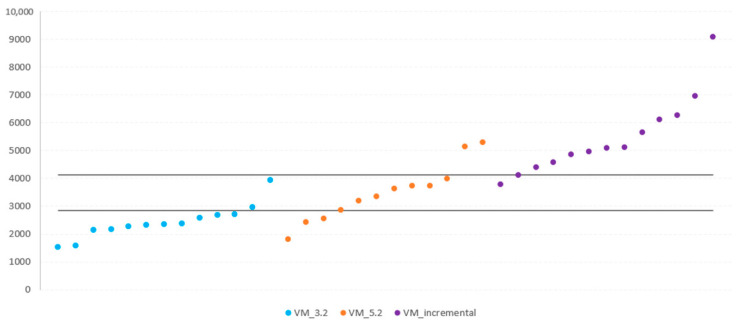
Vector Magnitude across the speed of 3.2 km/h, 5.2 km/h, and incremental test.

**Figure 2:**
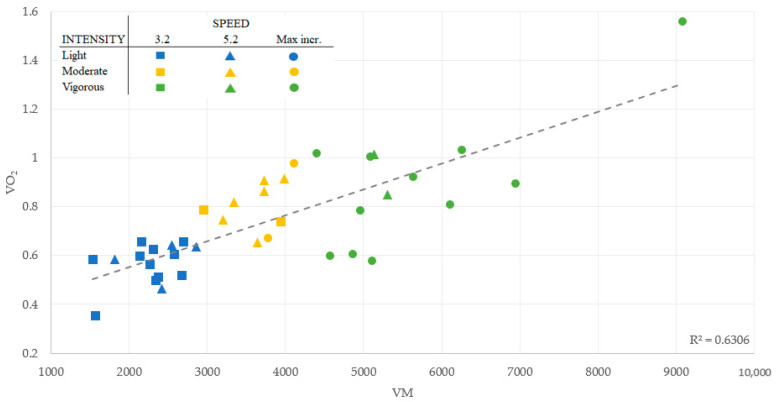
Linear Regression Model: Vector Magnitude association with the Relative Oxygen Consumption (VO_2_).

**Figure 3:**
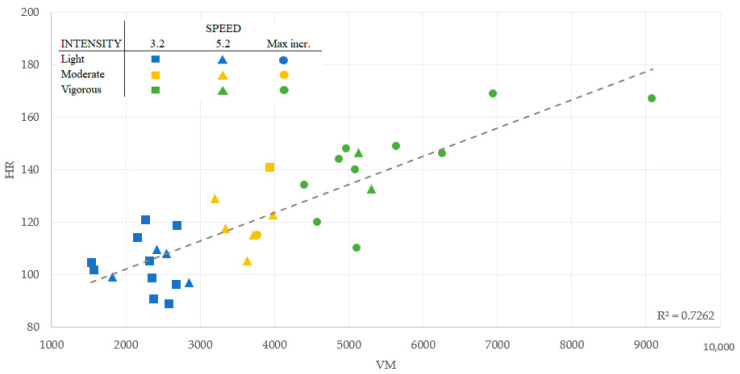
Lineal Regression Model: Vector Magnitude association with the Heart Rate.

Visual inspection of the distribution of the VM values (Figure 1) revealed that most VM values lay around the mean, with only a few points in extreme values across the walking speeds (3.2 km/h, 5.2 km/h, and incremental test), indicating high consistency of the data. Likewise, there was a moderate-high association between VM and VO_2,_ with an average r-squared (R^2^) value of 0.63 (Figure 2), and between VM and HR, with an average of R^2^ of 0.72 (Figure 3).

## 4. Discussion

To our knowledge, this is the first study to provide accelerometer cut-points for different walking speeds in adults with mild-moderate HD using two gold standards: indirect calorimetry and HR. We determined optimal VM cut-points for 3.2 km/h as ≤2852, 5.2 km/h from >2852 to ≤4117, and incremental speed as >4117.

Several studies have determined cut-points for physical activity in healthy populations [11,23,24,25]. In two studies [11,26], the mean cut-points for moderate intensity activity in healthy subjects were between 3208 and 8565 counts per minute and 2690 to 6167, higher than our results in HD with 2852 to 4117. In contrast, other studies reported similar cut-points to ours [11,27]. On the contrary, for PD, established VM cut-points ranged from 1881 to 2883 counts per minute for moderate-intensity activity [10,27]. Similarly, in subjects with coronary heart disease, moderate and vigorous physical activity cut-points ranged from 1800 to 3800 counts per minute, respectively [28], lower than our results in HD. Interestingly, in subjects with rheumatoid arthritis, similar cut-points to HD were found for moderate-intensity activity [29]. 

Although we do not have a compelling explanation for these discrepancies, we hypothesize possible explanations. Firstly, the influence of using different methodologies. In this regard, although we have followed standard methodologies and the cut-points were established by splitting the files into 1-min intervals [30,31], developing a consensus for data processing is essential to facilitate comparison between studies and extrapolate these findings to different conditions and settings.

Secondly, the importance of where the ActiGraph location is placed: chest, hip versus lower/upper extremities, limiting the applicability and interchange of these algorithms between different locations. Ideally, the accelerometer should be attached as close to the body’s center of mass as possible [32]. Thirdly, the performance on the treadmill test depends on the characteristics of the study population. It seems that diseases characterized by slowness, such as PD or rheumatoid arthritis, achieve moderate physical activity with lowers intensities. On the contrary, HD, characterized by hyperkinetic and hypokinetic movements, seems to obtain moderate activity with a higher level of physical activity but lower compared with healthy populations.

While the sample size was small, the strength of this investigation is its uniqueness; this is the first feasibility study using ActiGraph accelerometer cut-points for different walking speeds. We are aware that the results of this study cannot be extrapolated to patients with advanced HD due to their difficulties with independent walking, nor with different brands or models of accelerometers. For this first feasibility study, we have pre-established physical activity as light (walking speed of 3.2 km/h), moderate (walking speed of 5.2 km/h), and vigorous (incremental test) intensity, given the characteristics of our population. Further studies in larger HD samples should be conducted to compare our results, establishing unrestricted physical activity intensities using indirect calorimetry data. Likewise, our study was conducted in a laboratory under controlled conditions. Future studies should be conducted in free-living environments to understand real-life patient’s conditions by monitoring different activities across a continuum (i.e., moderate-vigorous, light physical activity, sedentary behavior, and sleep) during a 24-h day.

Based on the last recommendations of the WHO [1], there is an increased awareness about the need to implement physical activity interventions and decrease sedentary lifestyle behaviors. Adequate PA counseling could improve functional health and daily living activities, reducing sarcopenia and frailty in HD. These results provide valuable information and allow further research into the relationship between physical activity and HD progression.

## 5. Conclusions

This study provides accelerometer cut-points based on walking speed for physical-activity measurement in patients with mild to moderate HD. With the results of this study, we hope to provide the rationality for quantifying physical activity and increasing the knowledge about the impact of this intervention on HD progression.

## Figures and Tables

**Table 1:** Generated cut-points per walking speed intervals.

Axis	Speed ^a^	Sensitivity (%)	Specificity (%)	AUC (95% CI) ^b^	Cut-Point
VM	3.2	75	84.6	0.82051 (0.61639–0.94377)	≤2852
	5.2				2852–4117
	Incremental test	92.3	83.3	0.89103 (0.70174–0.97935)	>4117

Sensitivity, specificity, AUC, and cut-points in the vector magnitude (VM) for the defined walking speeds. ^a^ Walking speed in km/h; ^b^ Area under the curve; CI = confidence interval.
